# Cholera: Its Prevention and Treatment

**Published:** 1893-06

**Authors:** 


### BOOK NOTICES AND REVIEWS.

CHOLERA: ITS PREVENTION AND TREATMENT. By Elmer Lee, M. D. Reprint from *The Doctor of Hygiene* for April, 1893.

This small pamphlet sets forth one of the latest ideas in the dominant school of medicine for treating cholera successfully. The author says: "It is now well known that cholera is a disease of the alimentary canal. Its inciting cause is believed to be a germ taken into that canal through the medium of food and drink. There its presence is protested against by the absorbent vessels, which eliminate from the food the nutriment for the body. * * * * If the stomach could be emptied of the foul material before the poison has passed further, there might be speedy relief, and, indeed, no real cholera. * * * * A large irrigation (one to three gallons) of hot water made soapy preferably by neutral liquid soap introduced into the colon through a suitable rubber tube, is the simplest, and I am prepared to say further, the most satisfactory way of treating cholera. The time to begin the irrigation is at the very earliest possible moment. * * * For internal treatment, my experience has taught me that the medicinal peroxide of hydrogen of Marchand given in cupful doses of the strength of four per cent., or even stronger, is a better antiseptic than any other drug heretofore known in the treatment of cholera." Thus it will be seen that the latest teaching with regard to cholera is that it is a germ disease which must be treated by irrigation, to wash away the germs, and by peroxide of hydrogen to kill the germs that escape removal by irrigation.

The cholera excitement is subsiding, and we may express the hope that we may yet be spared a general visitation of this terrible scourge.

